# Tobacco and Liquor

**Published:** 1858-01

**Authors:** 


### TOBACCO AND LIQUOR.

THOSE who revel in these luxuries have an interesting time in prospect. It is stated, that in order to give an *almond flavor* to tobacco, the manufacturers are beginning to use prussic acid —a few drops of which on a man's tongue will produce death in five minutes. Several persons are alleged to have lost the use of their lower limbs by smoking cigars thus flavored.

A Government Inspector states, that of several hundred lots of liquor examined, nine-tenths were imitations, and that a great portion of them were poisonous concoctions. Not one gallon of brandy in a hundred is pure; and as to the wines, not one in a thousand; that chemical analysis shows them to be made of water, alum, pepper, horse-radish, and oil of vitriol; and that some of the whisky had enough of sulphuric acid in a quart to eat a hole in a man's stomach.

The Council of State, of *Berne*, Switzerland, in consequence of the deleterious effects of tobacco on the human frame, have recently determined to prohibit the use of it to all "unconfirmed" young men: this religious rite is there administered at sixteen.

A highly esteemed Presbyterion clergyman, in Virginia, rently committed suicide, from a state of nervous irritation, caused by the excessive use of tobacco.

An instructive and alarming fact may be here stated, in reference to the Wall-street forger, recently sent to the Penitentiary. It was proven on the trial, that he was never seen down town without having a cigar in his mouth; that he was never well. On entering the prison, smoking was absolutely and at once prohibited, by an inflexible rule. In three months he gained fifteen pounds in flesh, and his general health was improved in proportion. This showed the value of the expression "*I can't do it*," so readily used by slaves to the habit. No man who is a man will use that phrase in reference to any bodily habit. He who does it utters an unqualified untruth, and should be ashamed of himself, not only for his want of courage, but for his want of morality.

A large quantity of snuff was found lodged in the nasal cavities of the celebrated *Dr. Cooper*, of Boston, who was an inveterate snuff-taker, and died of a disorder of the head, induced by the pernicious habit.

*General Sullivan*, of the Revolutionary army, carried his snuff loose in his vest pocket. "At times," says the *Medical World*, "he had violent pains in the head; the intervals grew shorter, and the returns more distressing, ending in *palsy*, which rendered him helpless and miserable, and put him in his grave before he was fifty years old. *The earlier in life, and the earlier in the day tobacco is used, the more pernicious is its effect on the constitution.*

